# Vitamin D promotes the cisplatin sensitivity of oral squamous cell carcinoma by inhibiting LCN2-modulated NF-κB pathway activation through RPS3

**DOI:** 10.1038/s41419-019-2177-x

**Published:** 2019-12-09

**Authors:** Zixian Huang, Yin Zhang, Haigang Li, Yufeng Zhou, Qianyu Zhang, Rui Chen, Tingting Jin, Kaishun Hu, Shihao Li, Yan Wang, Weiliang Chen, Zhiquan Huang

**Affiliations:** 1 Guangdong Provincial Key Laboratory of Malignant Tumor Epigenetics and Gene Regulation, Sun Yat-Sen Memorial Hospital, Sun Yat-Sen University, Guangzhou, China; 2 Department of Oral and Maxillofacial Surgery, Sun Yat-sen Memorial Hospital, Sun Yat-sen University, Guangzhou, China; 3 Medical Research Center, Sun Yat-Sen Memorial Hospital, Sun Yat-Sen University, Guangzhou, China; 4 Department of Pathology, Sun Yat-sen Memorial Hospital, Sun Yat-sen University, Guangzhou, China; 5 State Key Laboratory of Oncology in South China, Sun Yat-Sen University Cancer Center, Guangzhou, China

**Keywords:** Cancer therapeutic resistance, Oral cancer

## Abstract

Chemoresistance is a major cause of cancer progression and the mortality of cancer patients. Developing a safe strategy for enhancing chemosensitivity is a challenge for biomedical science. Recent studies have suggested that vitamin D supplementation may decrease the risk of many cancers. However, the role of vitamin D in chemotherapy remains unknown. We found that vitamin D sensitised oral cancer cells to cisplatin and partially reversed cisplatin resistance. Using RNA-seq, we discovered that lipocalin 2 (LCN2) is an important mediator. Cisplatin enhanced the expression of LCN2 by decreasing methylation at the promoter, whereas vitamin D enhanced methylation and thereby inhibited the expression of LCN2. Overexpression of LCN2 increased cell survival and cisplatin resistance both in vitro and in vivo. High LCN2 expression was positively associated with differentiation, lymph node metastasis, and T staging and predicted a poor prognosis in oral squamous cell carcinoma (OSCC) patients. LCN2 was also associated with post-chemotherapy recurrence. Moreover, we found that LCN2 promoted the activation of NF-κB by binding to ribosomal protein S3 (RPS3) and enhanced the interaction between RPS3 and p65. Our study reveals that vitamin D can enhance cisplatin chemotherapy and suggests that vitamin D should be supplied during chemotherapy; however, more follow-up clinical studies are needed.

## Introduction

Oral cancer is one of the most lethal malignant tumours in the world, and its incidence is increasing yearly^1,2^. It occurs in sites highly vascularises with blood and lymphatic vessels, which may lead to in situ recurrence and distant metastasis. Hence, the prognosis of oral cancer patients is poor.

The major type of oral cancer is oral squamous cell carcinoma (OSCC). Cisplatin is the first-line drug for postoperative chemotherapy in OSCC patients; however, >30% of patients are initially insensitive to platinum-based chemotherapy, and the rest gradually become insensitive to previously effective drugs after several rounds of chemotherapy^3,4^. Intrinsic and acquired chemoresistance are major causes of the progression and mortality of oral cancer patients. Some strategies, such as combination chemotherapy and radiotherapy and the combination of different types of chemotherapeutic drugs, have been applied in patients. However, due to some common underlying mechanisms, chemo-resistant patients are usually insensitive to other therapy strategies^5^. Moreover, these combination therapy strategies could significantly increase the side effects of chemotherapy, such as bone marrow suppression and the impairment of immune cells in the tumour microenvironment^6,7^. Therefore, how to effectively overcome chemotherapy resistance while minimising adverse effects is a continuing challenge for biomedical science.

Vitamin D is a kind of fat-soluble secosteroid (also known as 1,25-dihydroxy-vitamin D3 (1,25(OH)2D3)). It mediates numerous physiological functions, such as regulating calcium and phosphorus metabolism, maintaining stable plasma calcium and phosphorus levels and participating in the growth of teeth and bones^8^. Clinically, it is often used for the treatment of rickets and osteoporosis.

In recent years, evidence from preclinical and clinical studies has suggested that vitamin D supplementation may decrease the risk of many cancers^9^.

Vitamin D has been found to have antitumour effects, suggesting that it might inhibit the progression of rectal cancer^10^, breast cancer^11^, pancreatic cancer^12,13^, head and neck cancers^14^ and other cancers. Urashima^15^ suggested that the higher the serum level of vitamin D is, the better is the prognosis of rectal cancer patients. However, the effects of vitamin D in chemotherapy remain unknown.

Here, we report that vitamin D could promote cisplatin sensitivity in OSCC cells and xenografted tumours. By analysing RNA-seq data in OSCC cells treated with vitamin D and cisplatin, we found that lipocalin 2 (LCN2) was an important modulator of cisplatin sensitivity regulated by vitamin D. Moreover, we discovered that cisplatin could induce demethylation of the LCN2 promoter and thus increase its expression, and vitamin D could reverse this process. Both loss-of-function and gain-of-function experiments revealed that LCN2 could decrease the sensitivity of cisplatin in OSCC cells and xenografted tumours. Mechanistically, LCN2 regulates nuclear factor kappa B (NF-κB) activation by binding to RPS3 and enhancing the interaction between RPS3 and P65. Furthermore, we demonstrated that LCN2 expression in OSCC patients could affect postoperative survival time and is associated with recurrence after chemotherapy.

## Results

### Vitamin D promoted cisplatin-based chemosensitivity in OSCC

**Fig. 1:**
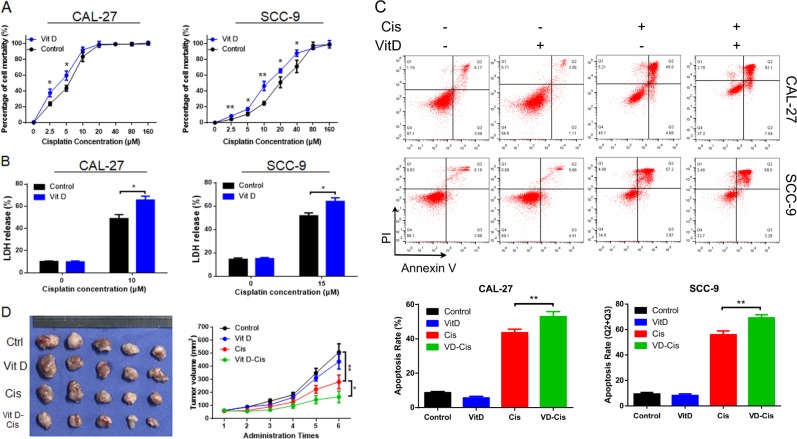
Vitamin D improved cisplatin chemosensitivity in OSCC. **a** After the pretreatment of oral cancer cells (CAL-27 and SCC-9) with vitamin D, a gradient concentration of cisplatin was applied to cells for 48 h, and MTS was used to detect absorbance at 492 nm; **b** LDH release assays were performed to determine the ratio of LDH to total LDH in cell supernatants after 48 h of vitamin D and cisplatin treatment; **c** Apoptosis rate of oral cancer cells after 48 h of vitamin D-cisplatin treatment determined by flow cytometry (annexin V/PI); **d** Oral cancer cell (CAL-27) xenograft tumour experiment results (tumour size statistics of different treatment groups).

To investigate the effects of vitamin D on cisplatin-based chemosensitivity, OSCC cells (CAL-27 & SCC-9) were pretreated with 30 nM vitamin D for 3 days and then treated with dose-gradient cisplatin^16^. MTS assay showed that vitamin D pretreatment resulted in the enhanced chemosensitivity of OSCC cells (Fig. 1a). Next, vitamin D-pretreated CAL-27 and SCC-9 cells were treated with 10 µM and 15 µM cisplatin, respectively. The cytotoxicity assay (lactate dehydrogenase (LDH) release, Fig. 1b) and flow cytometry (annexin V/PI, Fig. 1c) were used to detect cell death rates. Vitamin D had no significant effect on the OSCC cell death rate. However, in combination with cisplatin, it induced greater cell death than did cisplatin alone. Moreover, in CAL-27RE cells (the cisplatin-resistant cell line of CAL-27), vitamin D partially reversed cisplatin resistance (Fig. 1sA).

Subsequently, CAL-27 cells were used to establish a nude mouse xenograft model. When the xenograft grew to ~60 mm^3^, cisplatin and vitamin D were intraperitoneally injected, and the tumour volume was recorded (Fig. 1d). Although xenograft growth was slightly lower under vitamin D injection, the difference from control growth was not significant. Cisplatin inhibited the growth of tumours, and greater inhibition was observed when cisplatin was applied in combination with vitamin D. These data showed that vitamin D could enhance the sensitivity of OSCC to cisplatin and reduce cisplatin resistance.

### LCN2 was up-regulated after cisplatin treatment and down-regulated after vitamin D treatment

**Fig. 2:**
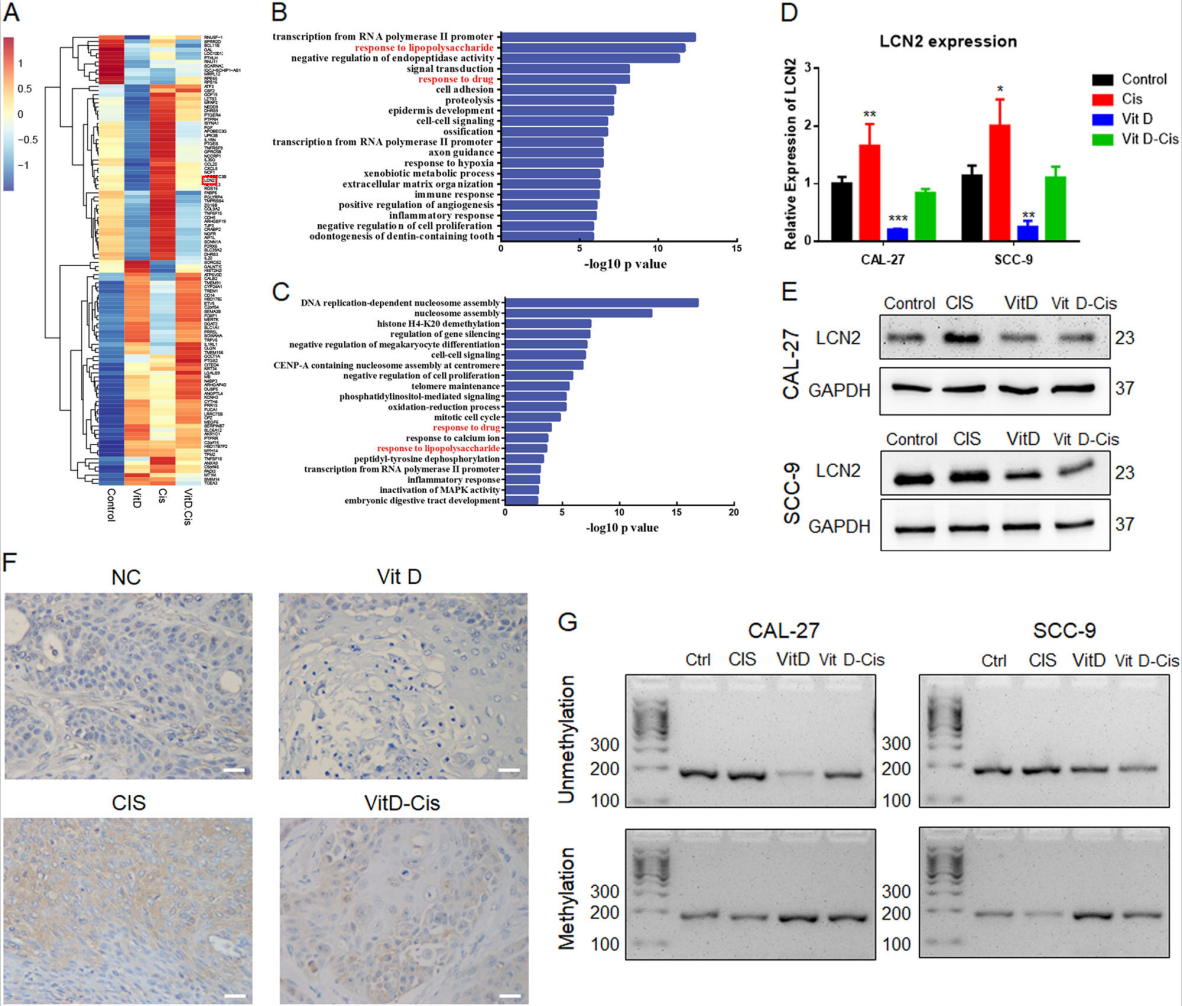
High-throughput sequencing analysis of differential LCN2 expression in a vitamin D-cisplatin chemotherapy model. **a** Heatmap of RNA-seq data in CAL-27 cells treated with PBS (control), vitamin D (Vit D), cisplatin (Cis) and vitamin D plus cisplatin (Cis + Vit D); **b** Gene Ontology (GO) analysis of differentially expressed genes in CAL-27 cells treated with vitamin D; **c** GO analysis of differentially expressed genes in CAL-27 cells treated with cisplatin; **d** qPCR detection of changes in the mRNA expression levels of LCN2 during vitamin D-cisplatin treatment; **e** Immunoblot analysis of the changes in LCN2 protein expression during vitamin D-cisplatin treatment; **f** Immunohistochemistry was used to detect the expression of LCN2 in the four groups of tumour tissues in nude mouse xenografts (scale bar: 20 μm); **g** Gel image of LCN2 promoter methylation and unmethylation.

To explore the mechanism by which vitamin D suppresses cisplatin resistance in OSCC, we pretreated CAL-27 cells with 5 µM cisplatin and 30 nM vitamin D for 36 h (Fig. 2sG). Then, total RNA was extracted for high-throughput sequencing. GO analysis showed the genes differentially expressed (DE) following vitamin D and cisplatin treatment shared some common biological process terms, such as response to lipopolysaccharide and response to drug (Fig. 2b, c). Many genes showed differential expression following treatment with vitamin D and cisplatin, in accordance with the phenotype in OSCC cells. Lipocalin-2 (LCN2) was one of the most significantly changed genes after both vitamin D and cisplatin treatment. When both vitamin D and cisplatin were administered, LCN2 expression differed little from that under control treatment, which revealed vitamin D could abrogate the up-regulation induced by cisplatin (Fig. 2a). LCN2, also known as oncogene 24p3 or neutrophil gelatinase-associated lipocalin (NGAL), belongs to the lipocalin family. Recently, LCN2 was reported to potentially play important roles in tumour progression and chemoresistance in many kinds of cancer^17^. Hence, we selected LCN2 for further analysis.

We verified the RNA-seq results by RT-qPCR and western blot, and similar results were obtained for both CAL-27 and SCC-9 cells (Figs. 2d, e and 1sF). We further analysed the expression of LCN2 in cisplatin-resistant cells. Compared with their expression in CAL-27 cells, LCN2 mRNA and protein expression was up-regulated in CAL-27RE cells (Fig. 1sB, C). We performed LCN2 immunohistochemical staining on the aforementioned OSCC xenografts (Fig. 2f and Table 1). The high-expression rate of LCN2 in tumour specimens after cisplatin treatment was 100% (5/5), while the control group and the vitamin D treatment group showed weak or no expression. When cisplatin was combined with vitamin D, the expression of LCN2 in the xenografts was partially inhibited (3/5). The trend in vivo was similar to that in vitro.

**Table 1:** Association of LCN2 expression with the features of Xenograft.

Group	LCN2 expression		*P* value
	High	Low	
*Without Vit D*			
Control	1	4	
Cisplatin	5	0	0.048
*Vit D applied*			
Control	0	5	
Cisplatin	3	2	0.167

Vitamin D receptor (VDR) is a ligand-inducible transcription factors. To investigate whether LCN2 is a target gene of VDR, we knocked down VDR by siRNAs. QPCR and western blot analysis revealed no pronounced changes after the successfully silencing of VDR (Fig. 2sD–F). These results imply that vitamin D regulates LCN2 expression via a VDR-independent mechanism.

Chemotherapy sensitivity may be due to changes in gene expression caused by epigenetic changes such as DNA methylation at the promoter after treatment^18^. Methylation at promoters has been reported to play an important role in regulating LCN2 expression^17^. So we evaluated methylation status after vitamin D and cisplatin treatment. Cisplatin treatment decreased the degree of methylation, whereas treatment with vitamin D increased the methylation of the LCN2 promoter. Moreover, vitamin D reversed the aberrant methylation caused by cisplatin, which ultimately reduced the expression of LCN2 (Fig. 2g). These results indicate that vitamin D and cisplatin regulate the expression of LCN2 by regulating LCN2 promoter methylation.

### LCN2 expression is associated with cisplatin insensitivity in OSCC cells

**Fig. 3:**
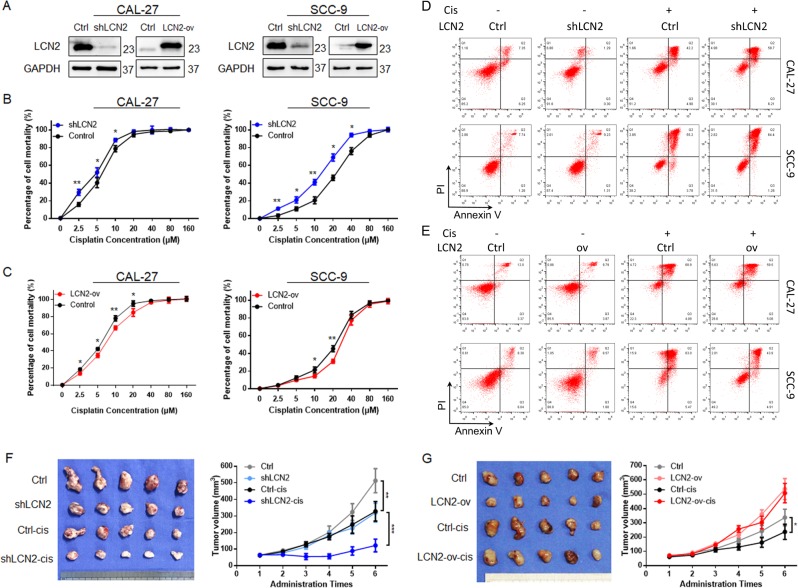
Regulation of the LCN2 gene on cisplatin sensitivity in oral cancer. **a** Immunoblot analysis clarified that LCN2 was successfully inhibited or overexpressed in OSCC cells; **b** Gradient concentration of the cisplatin-treated OSCC shLCN2 cell line (MTS assays were performed to determine the enhancement of cisplatin sensitivity); **c** Gradient concentration of the cisplatin-treated OSCC LCN2-ov cell line (MTS assays were performed to determine the inhibition of cisplatin sensitivity); **d** Flow cytometry detected the cisplatin effect on the OSCC shLCN2 cell line; **e** Flow cytometry detected the cisplatin effect on the OSCC LCN2-ov cell line; **f** Xenograft growth statistics from groups injected with the CAL-27 shLCN2 cell line; **g** Xenograft growth statistics from groups injected with the CAL-27 LCN2-ov cell line.

To investigate the relationship between the expression level of LCN2 and the effect of cisplatin on OSCC, we constructed LCN2-overexpressing cell lines (LCN2-ov) and knockdown cell lines (sh-LCN2) of CAL-27 and SCC-9 cells, which were verified by PCR (Fig. 3sA) and western blot analysis (Figs. 3a and 3sB). When LCN2 expression was down-regulated, OSCC cell cisplatin sensitivity was up-regulated (Fig. 3b). At a given concentration, cisplatin induced a high rate of death in sh-LCN2 cells (Figs. 3d and 3sC); when LCN2 was overexpressed, OSCC cells were significantly more resistant to cisplatin (Fig. 3c). Flow cytometry (Figs. 3e and 3sD) showed that the mortality of LCN2-overexpressing cells was decreased relative to that of shLCN2 cells at the same concentration of cisplatin. The down-regulation of LCN2 also inhibited the chemoresistance of CAL-27RE cells to cisplatin (Fig. 1sD). This finding indicated that LCN2 was an important regulator of cisplatin sensitivity in OSCC cells.

Next, two additional xenograft models were established using the CAL-27 LCN2 stable cell line (shLCN2 & LCN2-ov), and cisplatin chemotherapy was administered. The results showed that upon silencing LCN2 expression, tumour growth was inhibited (Figs. 3f and 3sE), which was in accordance with the in vitro experiments. After cisplatin treatment, both the control group and the shLCN2 group showed decreasing trends, but the latter group showed a greater decrease, indicating a higher cisplatin sensitivity of sh-LCN2 cells. The opposite trend was observed in LCN2-overexpressing xenografts. In the LCN2-ov group (Figs. 3g and 3sF) cisplatin chemotherapy had little effect on tumour size, whereas cisplatin significantly reduced the size of tumours in the control group.

These data reveal that LCN2 is an important regulatory target for tumour chemosensitivity during cisplatin therapy in OSCC and that the inhibition of LCN2 can significantly promote chemotherapy effects.

### Vitamin D suppressed NF-κB phosphorylation via LCN2 inhibition

**Fig. 4:**
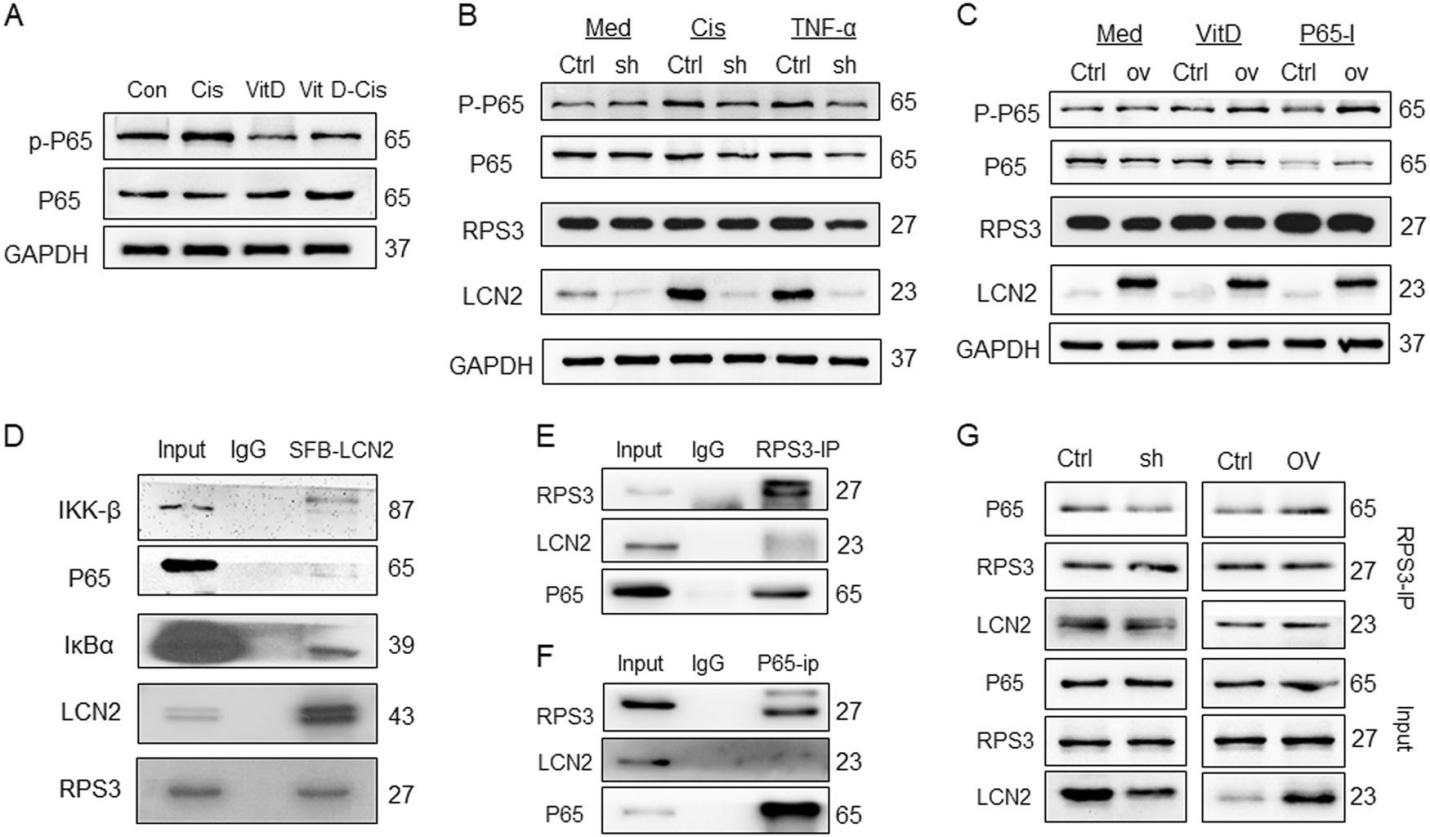
The LCN2-RPS3 complex inhibited P65 phosphorylation. **a** Immunoblot analysis of the effects of vitamin D and cisplatin treatment on NF-κB (P65) activation (p-p65); **b** After the inhibition of LCN2 expression, cisplatin and TNF-α stimulation had a weak effect on the expression of P-P65, whereas the expression of P-P65 in the control group was significantly increased. The treatment had little effect on the expression of RPS3; **c** After the upregulation of LCN2, vitamin D and NF-κB inhibitors (BAY-11-7085, P65-I in this legend) exhibited a decreased ability to inhibit P-P65 expression, and this treatment had little effect on RPS3 expression; **d** After the SFB tag was fused to LCN2, IP experiments were performed using an SFB tag, which revealed weak binding between LCN2 and the NF-κB pathway; **e** Endogenous RPS3-IP experiment: interaction between RPS3 and LCN2 and P65; **f** Endogenous NF-κB IP assay: NF-κB binds directly to RPS3 but not LCN2; **g** The RPS3-IP experiment was performed in LCN2 stable cell lines: the binding between RPS3 and P65 was decreased after LCN2 inhibition; the binding ability between RPS3 and P65 was enhanced after LCN2 expression was up-regulated. Med, culture medium; Cis, cisplatin; P65-I, p65 inhibitor.

The NF-κB pathway is a fundamental pathway in cancer. The constitutive activation of NF-κB has been demonstrated in various types of cancer. NF-κB can result in chemoresistance by activating many anti-apoptotic genes, such as FLIP, c-IAP1/2 and XIAP^19^. Our previous worked indicated that vitamin D could inhibit the NF-κB pathway^16,20^. Moreover, KEGG analysis of RNA-seq data from CAL-27 cells showed that cisplatin-induced genes were enriched in the NF-κB pathway (Fig. 4sD).

So we explored whether NF-κB play important roles in LCN2-mediated cisplatin resistance. We found that cisplatin stimulated the activation of NF-κB (enhanced P65-S536 phosphorylation), and vitamin D inhibited the phosphorylation of NF-κB; thus, the activation of p-p65 caused by cisplatin stimulation decreased in the VitD-cis group (Figs. 4a and 4sH). The vitamin D-NF-κB regulatory trend is consistent with LCN2, as shown in Fig. 2f. Therefore, we further speculate that LCN2 is an important regulator between vitamin D and NF-κB.

When LCN2 was inhibited, cisplatin or TNF-α stimulation slightly increased the phosphorylation level of p65, whereas the expression of p-p65 was enhanced in the control group (Figs. 4b and 4sI). Similarly, when LCN2 was overexpressed, p-P65 inhibition by vitamin D and the NF-κB inhibitor (P65-I, BAY-11-7085) was attenuated (Figs. 4c and 4sJ). Hence, LCN2 is a key protein in the regulation of NF-κB by vitamin D and cisplatin.

### The LCN2-RPS3 complex regulated P65 activation

To investigate the mechanism by which LCN2 regulates NF-κB, we first used an endogenous antibody for immunoprecipitation (IP) experiments. However, we did not detect an association between LCN2 and the NF-κB pathway (Fig. 4sA). Subsequently, we constructed an SFB tag in the LCN2 N-terminus and used it for IP experiments^21^, which revealed that LCN2 has a weak linkage with NF-κB signalling pathway protein (Fig. 4d). Therefore, we hypothesised that regulatory proteins function in interactions between LCN2 and the NF-κB pathway.

Next, we used mass spectrometry to identify the proteins involved in the interaction between LCN2 and NF-κB. Discovered by mass spectrometry (Fig. 4sE–G), an important NF-κB regulator, ribosomal protein S3 (RPS3)^22^, was found to bind to LCN2.

We verified the mass spectrometry result by IP and detected strong interactions of LCN2 and RPS3 through both SFB-tagged and endogenous antibody of LCN2 (Figs. 4d and 4SA). A direct interaction between RPS3 and the NF-κB pathway was detected (Figs. 4e, f, and 4SB), which has been reported previously^23^. Previous studies showed NF-κB activation is affected by the binding of RPS3 to P65^24^, so we speculated that LCN2 may interact with P65 indirectly: LNC2 may bind directly to RPS3 and promote the interaction between RPS3 and P65, thereby regulating NF-κB activation.

To test this hypothesis, we conducted co-immunoprecipitation (Co-IP) assays in LNC2-overexpression and knockdown stable cell lines (Fig. 4c). We found that upon silencing LCN2, the interaction between RPS3 and P65 was reduced; when LCN2 was overexpressed, RPS3 and P65 binding increased. Moreover, vitamin D and cisplatin treatment yielded no significant changes in RPS3 expression (Fig. 4sC). These data suggest that LCN2 could activate the NF-κB pathway by enhancing the interaction between RPS3 and P65.

### The expression of LCN2 is associated with the prognosis of OSCC patients

**Fig. 5:**
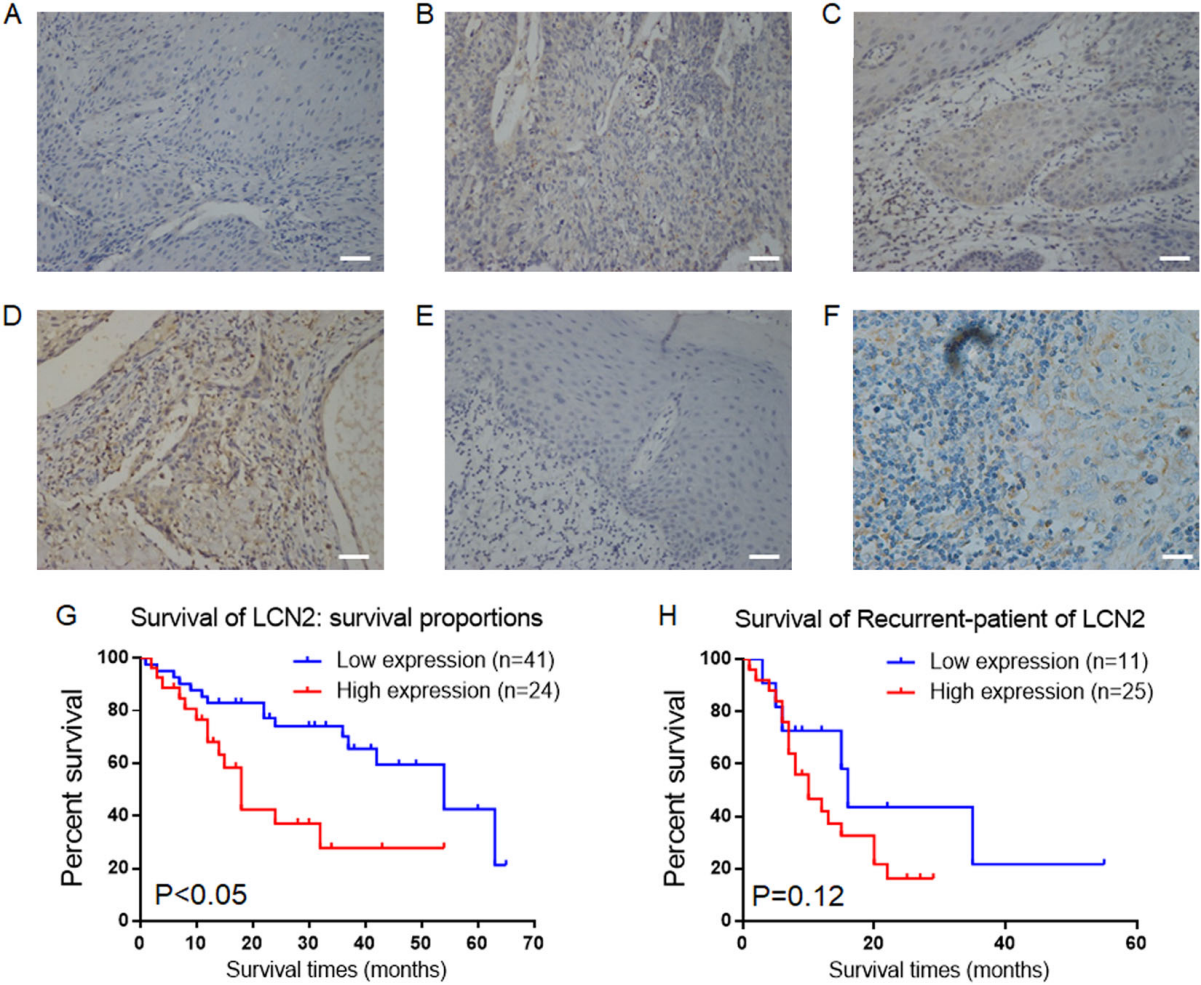
The expression of LCN2 is associated with the poor prognosis of patients with OSCC. **a** Immunohistochemical analysis of LCN2 expression (grade 0) in well-differentiated OSCC; **b** Immunohistochemical analysis of LCN2 expression (grade 1) in well-differentiated OSCC; **c** Immunohistochemical analysis of LCN2 expression (grade 2) in moderately differentiated OSCC; **d** Immunohistochemical analysis of LCN2 expression (grade 3) in poorly differentiated OSCC; **e** Negative LCN2 expression in ANC tissue; **f** Positive LCN2 expression in lymphatic metastases; **g** Kaplan–Meier estimates of the overall survival of patients with LCN2 expression; **h** Kaplan–Meier estimates of the overall survival of patients with LCN2 expression who experienced recurrence (scale bar: 20 μm).

We performed LCN2 immunohistochemical staining analysis on specimens from patients with OSCC who were not previously treated with radiotherapy or chemotherapy (Table 2 and Fig. 5a–f). The high-expression rate of LCN2 was low (36.9%, 24/65) in this group, and LCN2 expression did not correlate with patient age or gender but was positively correlated with lymph metastasis, clinical stage (T stage) and malignancy (differentiation). Patients with OSCC who had high LCN2 expression experienced a shorter survival time (Fig. 5g). Hence, LCN2 is one of the potential indicators of a poor prognosis in clinical patients.

**Table 2:** Association of LCN2 expression with the features of OSCC patients.

Characteristics	LCN2 expression		*P* value
	High (%)	Low (%)	
*Age*			
≤40	11 (42.31)	15 (57.69)	
>40	13 (33.33)	26 (66.67)	0.4705
*Gender*			
Male	14 (36.84)	24 (63.16)	
Female	10 (37.04)	17 (62.96)	0.9874
*Differentiation*			
Well	6 (22.22)	21 (77.78)	
Moderate	8 (36.36)	14 (63.64)	
Poor	10 (62.50)	6 (37.50)	0.0087
*Lymphatic metastasis*			
No	7 (21.88)	25 (78.12)	
Yes	17 (51.52)	16 (48.48)	0.0128
*Clinical stage (T)*			
1	2 (11.76)	15 (88.24)	
2	9 (40.91)	13 (59.09)	
3	8 (50.00)	8 (50.00)	
4	5 (50.00)	5 (50.00)	0.0233

Lcn2 positive 24 (36.92%), negative 41 (63.08%)

**Fig. 6:**
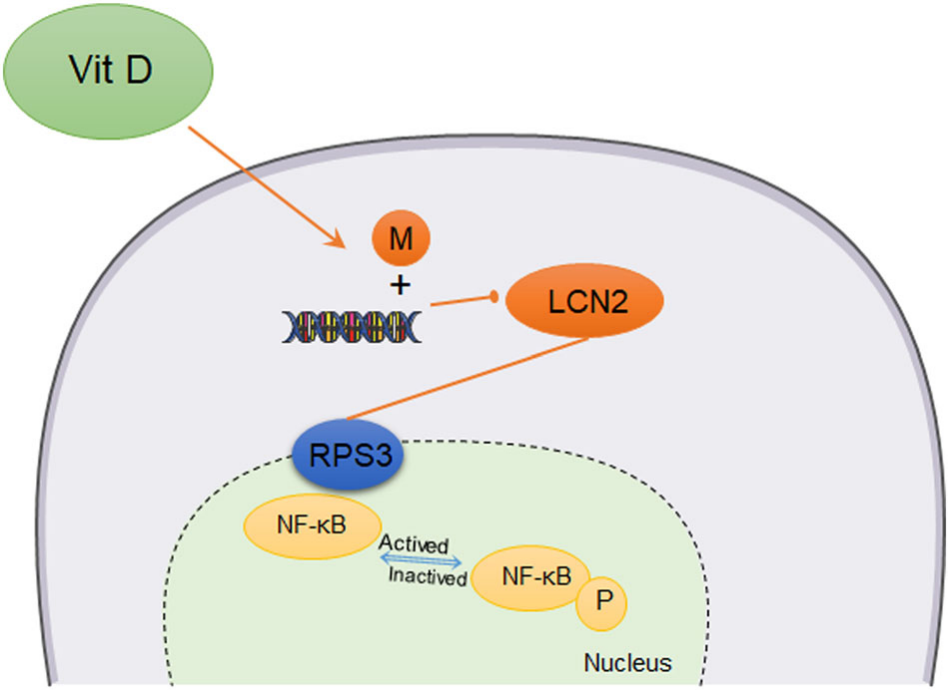
Schematic model of how vitamin D promotes the cisplatin sensitivity of oral cancer by inhibiting the LCN2-RPS3-NF-κB pathway. In OSCC cells, vitamin D promoted LCN2 promoter methylation to inhibit LCN2 protein expression, which reduced LCN2-RPS3 binding and thus reduced RPS3-NF-κB binding. The formation of the NF-κB complex ultimately reduced the activation of NF-κB, increasing the chemotherapeutic sensitivity of the tumour to cisplatin.

Is LCN2 expression related to recurrence after chemotherapy? To address this question, we collected samples from 36 patients with recurrent OSCC and performed immunohistochemical staining. The high-expression rate of LCN2 in recurrent patients was 69.4% (25/36). Among them, 10 patients with recurrence could track their primary tumour samples: the initial rate of LCN2 was 60.0% (6/10), and the recurrence rate of LCN2 was as high as 80.0% (8/10). Low-expression LCN2 patients had elevated expression after chemotherapy; two patients with low LCN2 expression did not receive chemotherapy; two patients had high LCN2 expression and did not experience recurrence after chemotherapy; and four patients had high LCN2 expression and relapsed after chemotherapy.

Among the relapsed patients, 20 patients were treated with chemotherapy, and 17 patients were positive for LCN2 after recurrence (85.0%, 17/20). The correlation analysis confirmed that in patients with relapse, high LCN2 expression was associated with chemotherapy (Table 3), and chemotherapy induction promoted high LCN2 expression. There was a difference in LCN2 expression between relapsed patients and primary patients (Table 4). However, in patients with recurrence, there was no significant correlation between the expression of LCN2 and survival time (Fig. 5h), which may be related to the small sample size. Further research needs to expand the sample size to more clearly define the relationship between LCN2 expression and survival in oral cancer patients with recurrence.

**Table 3:** Differential expression of LCN2 in patients with primary or recurrent OSCC.

Characteristics	LCN2 expression		*t*	*P*-value
	High	Low		
*OSCC patients*				
Primary	24	41		
Recurrent	25	11	3.263	0.002

**Table 4:** Correlation between LCN2 and cisplatin-chemotherapy in recurrent samples.

Characteristics	LCN2 expression		*R*^2^	*P*-value
	High	Low		
*Chemotherapy*				
Yes	17	3		
No	8	8	0.143	0.023

In conclusion, LCN2 was a key molecule responsible for drug sensitivity during cisplatin chemotherapy in OSCC. Vitamin D inhibited NF-κB activation by suppressing LCN2, which enhanced the cisplatin chemotherapeutic effects on OSCC (Fig. 6).

## Discussion

The antitumour effect of vitamin D is a popular research topic. Epidemiological studies have compared the relationship between serum vitamin D levels and prognosis in patients with breast cancer^25^ and rectal cancer^26^ and have found that the higher the serum level of vitamin D is, the better is patient prognosis. The regulatory relationship between vitamin D and tumours has not yet been elucidated, and its mechanism has not been clarified. Vitamin D enters cells by two pathways: the steroid receptor pathway and the direct entry pathway. Vitamin D receptor (VDR) is a membrane receptor for vitamin D. However, whether VDR is a tumour suppressor or an oncogene remains unclear^27–29^. The VDR expressions in cisplatin and vitamin D treatments failed to show significant changes (Fig. 2sA–C). Hence, the disputed effects of VDR and vitamin D on cancer cells led us to postulate that the antitumour function of vitamin D is not solely borne by VDR and that other molecules are present.

In this study, we identified LCN2 as a potential regulator of vitamin D. LCN2, also known as neutrophil gelatinase-associated lipocalin (NGAL), is a tumour-promoting gene^30^. LCN2 expression regulates the cisplatin sensitivity of OSCC. In the vitamin D-cisplatin chemotherapy model, cisplatin promoted LCN2 expression, whereas vitamin D inhibited LCN2 expression and partially reversed cisplatin-induced LCN2 upregulation. The tumour xenograft model showed that vitamin D inhibited the expression of LCN2 in the tumour and enhanced cisplatin sensitivity, while the tumour with high LCN2 expression was not sensitive to cisplatin. Clinically, LCN2 was positively associated with differentiation, lymph node metastasis, T staging and a poor prognosis in OSCC patients. Patients with postoperative recurrence, especially those who relapsed after chemotherapy, exhibited a statically significant upregulation of LCN2. This finding was validated with our laboratory findings, clarifying that LCN2 is a tumour chemosensitive gene and has potential as a predictor of tumour recurrence^31^. Furthermore, we experimentally proved that the regulation of LCN2 and VDR genes is independent of each other. VDR inhibition had no significant effect on VDR expression. In summary, we believe that the antitumour regulation of vitamin D is attributed to the inhibition of LCN2.

However, how does vitamin D suppress LCN2 expression? Chemotherapy sensitivity may be due to changes in gene expression caused by epigenetic changes, such as DNA methylation at the promoter during treatment^32^. Cisplatin kills cancer cells by binding to DNA and inducing DNA damage. Recent studies have revealed that mismatch repair proteins can recruit DNA methylation enzymes at the DNA damage site and change the methylation status^33^. A reversal of the aberrant methylation of key cisplatin resistance-related genes may provide a novel solution to chemotherapy resistance. In this study, vitamin D promoted LCN2 promoter methylation and reduced LCN2 expression. Furthermore, the unmethylation of LCN2 was associated with malignant tumour characterisation and a poor prognosis, which may have resulted from the antitumour effect of vitamin D.

Vitamin D can regulate a variety of important pathways^34–37^. Among these, the NF-κB pathway plays an important role in the regulation of cisplatin sensitivity. NF-κB is present in almost all cells and has five family proteins^38,39^: RelA (p65), RelB, c-Rel, p50 and p52. The most common RelA (p65) C-terminus has a transactivation domain (TD) that activates the target gene. The sustained activation of NF-κB, represented by P65 phosphorylation, is an important mechanism for tumourigenesis, drug resistance regulation, and tumour self-defence^40^. The inhibition of NF-κB activation is beneficial to the treatment of tumours^41^. In this study, we found that once LCN2 was inhibited, the effect of NF-κB activation by cisplatin or TNF-α was weakened. When LCN2 was up-regulated, the inhibition of NF-κB by vitamin D or the NF-κB inhibitor was also attenuated.

These observations led to the question of whether there an intermediate regulatory factor between LCN2 and NF-κB or whether LCN2 acts directly on transcriptional activation^42^. When the IP assay was performed using an endogenous LCN2 antibody and SFB-fusion-LCN2, weak binding of LCN2 to NF-κB was observed, suggesting a regulatory factor between LCN2 and NF-κB. Mass spectrometry revealed that RPS3 may be the key to regulation. RPS3, which is located upstream of the NF-κB regulatory gene, activates the NF-κB pathway by interacting with the NF-κB complex^43^. The interaction among RPS3, LCN2 and NF-κB is essential for subsequent regulation. When LCN2 expression was decreased, it affected the binding between RPS3 and NF-κB, thereby reducing the activation of NF-κB.

A new vitamin D-LCN2-RPS3-NF-κB regulatory model was established. Vitamin D promoted LCN2 promoter methylation to inhibit LCN2 protein expression, reduced LCN2-RPS3 binding, and thus reduced RPS3-NF-κB binding. The formation of the NF-κB complex ultimately reduced the activation of NF-κB, thereby increasing the chemotherapeutic sensitivity of the tumour to cisplatin.

In conclusion, the present study identified a new vitamin D subregulator, LCN2, that could enhance the cisplatin effect on OSCC by inhibiting NF-κB activation. The downregulation of LCN2 was capable of promoting OSCC chemosensitivity in vitro and in vivo. More importantly, high LCN2 expression predicted a poor prognosis in OSCC patients and was associated with OSCC chemosensitivity and postoperative recurrence. Therefore, LCN2 could be used as a potential target for tumour chemosensitivity. At the same time, based on this study, the use of vitamin D is important for the inhibition of LCN2 and other genes, which benefited the sensitisation of tumour cells to chemotherapy. Although the initial expression of the LCN2 protein was low, the subsequent increase caused by chemotherapy cannot be ignored. Once LCN2 was highly expressed, the vitamin D inhibitory effect on LCN2-NF-κB was weakened, and the chemosensitivity of tumour cells was reduced. Therefore, vitamin D should be taken after the start of chemotherapy, but more follow-up clinical studies are needed to support this hypothesis.

## Materials and methods

### Antibodies

Mouse monoclonal anti-FLAG (#F3165) was purchased from Sigma. Antibodies against p-P65 (#3033), P-65 (#8242), RPS3 (#9538), IKK-β (#8943), IκBα (#4814) and GAPDH (#D16H11) were purchased from Cell Signaling Technology. VDR (#MABS2028) and LCN2 (#AB2267) antibodies were purchased from Millipore. Secondary antibodies, including goat anti-mouse IgG-HRP (sc-2005) and goat anti-rabbit IgG-HRP (sc-2004), were purchased from Santa Cruz Biotechnology.

### Reagents

The vitamin D metabolite 1,25D3 (Sigma, USA) was dissolved at a concentration of 400 μM in anhydrous alcohol (AA) for preservation. Immediately prior to use, the stock was diluted to a final concentration of 30 nM in culture medium. An NF-κB inhibitor (BAY 11-7082) was purchased from Beyotime (Shanghai, China). Other chemicals were purchased from Sigma. All of the culture media (DMEM for CAL-27/27RE, DMEM-F12 for SCC-9) and foetal bovine serum (FBS) were purchased from Bioind.

### Plasmids

Human LCN2 and LCN2 short hairpin RNAs (shRNAs) were purchased from Vigene Biosciences, and the sequence of the shRNA was as follows:

GAGCTGACTTCGGAACTAATTCAAGAGATTAGTTCCGAAGTCAGCTCTTTTTT. pMD2.G (#12259, Addgene) and psPAX2 (#12260, Addgene) were used as packaging vectors.

To generate overexpression cell lines, the LCN2 sequence was inserted into the pDonor vector and transferred into the pKO vector using previously described methods^21^. The FLAG and SFB tags were added in front of the N-terminus of LCN2.

All of the constructs were confirmed by both DNA sequencing and diagnostic digestion.

### Cell culture and transfection

The human oral squamous cell carcinoma cell lines CAL-27 and SCC-9 and the lentivirus vector packaging cell line HEK293t were obtained from the American Type Culture Collection (ATCC). The CAL-27RE cell line, a resistant strain of the CAL-27 cell line, was gifted from Professor Li (Sun Yat-sen Memorial Hospital, Sun Yat-sen University, China). All OSCC cell lines were routinely cultured in DMEM and DMEM-F12 medium supplemented with 10% FBS in a 37-°C humidified incubator containing 5% CO_2_. All the cell lines were validated by short tandem repeat profiling analysis and were free of mycoplasma contamination.

The transient transfection of OSCC cells was performed using Lipofectamine 3000 (Invitrogen) reagent according to the manufacturer’s instructions.

For transient transfections using small interfering RNAs (siRNAs), siRNAs targeting VDR, RPS3 and LCN2 were synthesised by GenePharma (Suzhou, China). The transfection was performed with Lipofectamine 3000 reagent (Invitrogen, Carlsbad, USA) according to the manufacturer's protocol. The siRNA sequences are shown as follows:

siRNA sequences for LCN2, VDR and RPS3

**Table:** 

Gene		Forward	Reverse
RPS3	s1	CCAGGACAGAAAUCAUUAUTT	AUAAUGAUUUCUGUCCUGGTT
	s2	GGUUGUGGUGUCUGGGAAATT	UUUCCCAGACACCACAACCTT
LCN2	s1	CCUCCGUCCUGUUUAGGAATT	UUCCUAAACAGGACGGAGGTT
	s2	GAGCUGACUUCGGAACUAATT	UUAGUUCCGAAGUCAGCUCTT
VDR	si	CCUGCUCAGAUCACUGUAUTT	AUACAGUGAUCUGAGCAGGTT

For stable expression, lentiviral plasmids harbouring the desired gene were first transfected into 293T cells together with the packaging plasmids pSPAX2 and pMD2.G at a ratio of 5:3:2. HEK293 cells were placed into a 10-cm plate and cultured as previously described. After reaching 70–80% confluence, the cells were transfected with 6 µg psPAX2, 3 µg pMD2.G and 10 µg transfer vector using Lipofectamine 3000 reagent. Forty-eight hours after transfection, the supernatants of each group were collected and used to infect OSCC cells for another 48 h. Puromycin-tolerant OSCC cells were picked. Subsequent western blotting and PCR were applied to confirm the correct expression of the stable cell lines.

### OSCC sample collection and patient follow-up

To address the research purpose, patients presenting at the Department of Oral and Maxillofacial Surgery, Sun Yat-sen Memorial Hospital between 2011 and 2013 for the treatment of OSCC were recruited. Inclusion criteria included a pathological diagnosis of OSCC and willingness to participate in the subsequent follow-up. Patients were excluded as study subjects if they were diagnosed with multiple cancer or other severe diseases. Data on the features of the OSCC patients, including age, gender, tumour differentiation, lymphatic metastasis and clinical stage, were collected. All patients would have a referral at least every season. In addition, their tumour samples and adjacent noncancerous (ANC) samples were collected. The ANC tissue refers to an area at least 2 cm from the tumour lesion, representing the resection border, being pathologically confirmed as noncancerous tissue.

### In vivo tumour xenograft and drug application

To explore the effects of vitamin D on OSCC tumours in vivo, CAL-27 cells and their LCN2-stable cell lines were applied for tumour xenografts. OSCC cells (2.0 × 10^6^) were implanted into the right upper backs of BALB/c nude mice (female, 5 weeks old). The tumours were measured every 3–4 days to determine the tumour volume (mm^3^) calculated as length × width^2^ × 0.5. The tumours reached ~5 × 5 mm (length × width, 60 mm^3^) after 3 weeks.

When the tumour volume reached ~60 mm^3^, 30 µg/kg 1,25D3 or 5 mg/kg cisplatin was intraperitoneally injected according to the treatment group. Based on the pre-experimental results in vitro, vitamin D was supplied first, and then chemotherapy was initiated. The vitamin D administration interval was 2 days, and the chemotherapy administration interval was 4 days. Tumour volume was measured before each chemotherapy event.

Tumour formation was observed, and tumour growth curves were constructed. The mice were killed after 5 injections, and the tumours were harvested and then frozen or paraffin embedded for immunohistochemical detection.

### Immunohistochemistry (IHC)

Immunohistochemical staining was performed according to standard protocols. After deparaffinization, antigen retrieval was conducted using 10 mM sodium citrate buffer (pH 8.0) in a pressure cooker at full power for 5 min. Briefly, the tissue sections were blocked sequentially with 3% H_2_O_2_ and normal serum and then incubated with primary antibodies at 4 °C overnight. The tissue sections were incubated with a biotinylated secondary antibody and conjugated with a streptavidin-HRP complex (ready-to-use SP kit; Zhongshan Co., Beijing, China). Finally, the sections were visualised with 3-3′-diaminobenzidine and counterstained with haematoxylin and mounted. The samples were rinsed with phosphate-buffered saline (PBS) between each step.

### Evaluation of IHC staining

IHC tissue staining was evaluated as previously described^44^ by 2 pathologists, who assessed the number of positive cells and the intensity of staining. The positive results were judged by semi-quantitative points. The staining intensity scores were 0 (negative), 1 (weak), 2 (medium) and 3 (strong). The percentage of positive cells was scored as 0 (0%), 1 (1–25%), 2 (26–50%) and 3 (>50%). The staining intensity score and the proportional score were added to obtain the total score. A total score ≥3 was considered to represent high expression. A total score <3 was considered to represent low expression.

### Western blot, immunoprecipitation and mass spectrometry

For protein extraction, the cells were washed twice with cool PBS, harvested by scraping and then lysed in lysis buffer (Beyotime, China). Following centrifugation, the supernatant was collected, and the protein concentration was determined using the BCA Protein Assay Kit (Pierce^TM^, USA).

For western blotting, cell lysates were electrophoretically separated on an SDS-PAGE gel using a standard protocol. The proteins were then transferred to polyvinylidene fluoride (PVDF) membranes (IPVH00010; Millipore, USA). The membranes were blocked with 5% non-fat milk in Tris-buffered saline containing 0.1% Tween-20 (TBST) for 1 h at room temperature. The blots were then incubated with the antibodies mentioned above at 4 °C overnight, washed in TBST and then probed with secondary antibody. Western blot analysis was performed using the grey value statistics of the blots.

For immunoprecipitation, the supernatants were first incubated with S-protein agarose beads (#69704, Millipore, for SFB-LCN2) overnight at 4 °C, and the precipitates were washed three times with NETN buffer. To detect endogenous interactions, the clarified supernatants were incubated with the antibodies mentioned above for 2 h and then with magnetic beads (Pierce™ Protein A/G Magnetic Beads, # 88802, Thermo Fisher overnight. After being washed three times with NETN buffer, the samples were collected and analysed by western blot.

Proteins immunoprecipitated by LCN2 antibody were digested, and the peptides were analysed by mass spectrometry.

### RNA extraction, real-time quantitative RT-PCR and RNA sequencing

Total RNA was extracted using TRIzol reagent (Takara, Japan) according to the manufacturer's instructions and then reverse transcribed into cDNA using the PrimeScript™ RT Master Mix (Takara, Japan) on an ABI 9700 Real-Time PCR system (ABI, USA). The newly synthesised cDNA was then used as a template for the detection of the desired gene.

Specifically, 1 μl of cDNA was mixed with TB Green® Premix Ex Taq™ II (Takara, Japan) in a 20-μl reaction. All of the reactions were run in triplicate using the primers described above. The reaction conditions were as follows: 94 °C for 2 min, 94 °C for 20 s, 58 °C for 20 s and 72 °C for 20 s, for 40 cycles. The relative expression of mRNA was detected using the Roche LightCycler 480 II Real-time PCR machine (Roche, USA). The primer sequences were as follows:

Primer sequences for PCR

**Table:** 

Gene	Forward	Reverse
LCN2	CCCGCAAAAGATGTATGCCA	TCTTAATGTTGCCCAGCGTG
VDR	GTGGACATCGGCATGATGAAG	GGTCGTAGGTCTTATGGTGGG
GAPDH	GAGTCAACGGATTTGGTCGT	GACAAGCTTCCCGTTCTCAG
RPS3	AGAGGAAGTTTGTCGCTGATG	GCACCTCAACTCCAGAGTAGC

The cells of the CAL-27 cell line were pretreated with 30 nM vitamin D for 3 days and then with 5 µM cisplatin and/or 30 nM vitamin D for 36 h. RNA was extracted using TRIzol (Thermo Fisher Scientific). After removing rRNA, RNAs were fragmented and reverse transcribed using random primers. cDNAs were ligated with adaptors, amplified via PCR and then sequenced with an Illumina sequence analyser. Filtered sequences were aligned with HISAT, and gene expression was analysed by DEGseq software. The differentially expressed genes were subjected to Gene Ontology (GO) and KEGG pathway analysis and were used to create heatmaps with the pheatmap package of R.

### DNA isolation, bisulfite treatment and methylation-specific PCR

Genomic DNA was isolated from OSCC cell samples with a DNA isolation kit (#3101050, Simgen, China). The bisulfite treatment of DNA was performed by using an EZ DNA Methylation-Gold^TM^ Kit (#D5055, ZYMO Research, USA) according to the manufacturer’s instructions.

### Quantitative methylation-specific PCR

Quantitative methylation-specific PCR (qMSP) was performed as described previously^31^. Briefly, genomic DNA was isolated with a Genomic DNA Purification Kit (Thermo Fisher Scientific, Cat. #K182104A) according to the manufacturer’s methods. For each sample, 0.8 µg of genomic DNA was treated with sodium bisulfite using the EZ DNA Methylation-GOLD Kit (ZYMO Research, USA, Cat. #D5008) following the manufacturer’s instructions. The resulting genomic DNA was subjected to PCR analysis with the following primers:

LCN2-methylated primer sequences

**Table:** 

LCN2		
Methylated	Forward	CGAGAGTTATTGCGTTTAGTCGA
	Reverse	CGAATAAATCACGAAATCAAAAATTCGA
Unmethylated	Forward	AGAGTTATTGTGTTTAGTTGAGGA
	Reverse	CAAATAAATCACAAAATCAAAAATTCAA

Methylation-specific PCR (MSP) was performed according to the MSP Kit (#R100A, Takara, Japan) protocol. Specificity, 2 μg of genomic DNA was used for sodium bisulfite treatment, and two sets of primers specific to the methylated and unmethylated target sequences were used in two PCRs. PCR products (a 273-bp product, both methylated and unmethylated) were separated by 3% agarose gel electrophoresis.

### Flow cytometry

For the flow cytometric quantification of cell death, the cells were treated as indicated. Then, cells were collected, washed twice with PBS and stained using Annexin V-FITC/PI (#556547, BD) according to the manufacturer’s instructions. Stained cells were analysed using a Becton Dickinson FACScan Flow Cytometer (FACScan, BD), and data were processed using FlowJo software.

### Cytotoxicity assay

Relevant cells were plated into 96-well plates (5000 cells/well) and cultured 24 h before treatment. Then, cell death was measured at the indicated time points with the LDH assay using a CytoTox 96 Non-Radioactive Cytotoxicity Assay Kit (#G1781, Promega) according to the manufacturer’s protocol.

The LDH release rates were determined following the protocol of the Cell-Mediated Cytotoxicity Assay kit:

$$\% {\mathrm{Cytotoxicity}} = \frac{{{\mathrm{Experimental}} - {\mathrm{Effector}}\,{\mathrm{spontaneous - Target}}\,{\mathrm{spontaneous}}}}{{{\mathrm{Target}}\,{\mathrm{maximum}} - {\mathrm{Target}}\,{\mathrm{spontaneous}}}} \times 100$$

### Cell proliferation assay

At 24 h after transfection, the cells were collected, and 2000 cells/well were plated into 96-well plates. The number of cells at 24, 48 and 72 h was determined using the MTS Assay Kit (#G3580, Promega, USA). The medium was removed from each well, and 100 μl of 10% MTS in DMEM was added. The plates were incubated for an additional 2 h, and absorbance at 490 nm was measured using a microplate reader (Multiskan MK3, China). The data were presented as original OD values.

### Cisplatin cytotoxicity assay

At 24 h after transfection, OSCC cells were seeded at 10,000 cells/well into 96-well plates. After being incubated overnight, the cells were treated with one of various concentrations (0, 2.5, 5, 10, 20, 40, and 80 μmol/L) of cisplatin (#1134357, Sigma, USA), and the MTS assay was applied to examine the cytotoxicity of cisplatin after 48 h of treatment.

The relative cell survival (%) was determined by the formula (OD × χμmol/L/OD 0 μmol/L) × 100, where χ represents cisplatin concentration.

The percentage of cell mortality (%) was then calculated as 100 - relative cell survival.

### Statistical analyses

All statistical analyses were conducted using SPSS 19.0 statistical software. Spearman correlation analyses were used to examine the correlation between LCN2 expression and cisplatin chemotherapy in patients who experience recurrence. An unpaired *t* test was used to compare LCN2 expression in patients with primary or recurrent OSCC tissues. Kruskal–Wallis analysis was used to examine the relationships between clinicopathological characteristics and protein expression. The survival curves were plotted using the Kaplan–Meier method and compared with the log-rank test. Fisher’s exact test was used to examine the relationships between LCN2 expression and xenograft features. Student’s *t* test was used to compare the PCR results, cell apoptosis, tumour xenograft results, and cell functions (proliferation, migration, invasion, etc.) between the different groups. Unless otherwise noted, quantitative data are expressed as the mean and standard error of the mean (S.E.M.). Statistical significance was determined with a paired Student’s *t* test. *P < 0.05; **P < 0.01; ***P < 0.001, compared with the control.

## Supplementary information

Supplementary merged

Supplementary figure 1s

Supplementary figure 2s

Supplementary figure 3s

Supplementary figure 4s

Supplementary figure 5s

## Data Availability

The datasets used for the current study are available from the corresponding author on reasonable request. All data generated or analysed during this study are included in this published article or its supplementary information files (Figs. 1s–5s).
