# Correction: Vitamin D promotes the cisplatin sensitivity of oral squamous cell carcinoma by inhibiting LCN2-modulated NF-κB pathway activation through RPS3

**DOI:** 10.1038/s41419-020-2389-0

**Published:** 2020-03-17

**Authors:** Zixian Huang, Yin Zhang, Haigang Li, Yufeng Zhou, Qianyu Zhang, Rui Chen, Tingting Jin, Kaishun Hu, Shihao Li, Yan Wang, Weiliang Chen, Zhiquan Huang

**Affiliations:** 1 Guangdong Provincial Key Laboratory of Malignant Tumor Epigenetics and Gene Regulation, Sun Yat-Sen Memorial Hospital, Sun Yat-Sen University, Guangzhou, China; 2 Department of Oral and Maxillofacial Surgery, Sun Yat-sen Memorial Hospital, Sun Yat-sen University, Guangzhou, China; 3 Medical Research Center, Sun Yat-Sen Memorial Hospital, Sun Yat-Sen University, Guangzhou, China; 4 Department of Pathology, Sun Yat-sen Memorial Hospital, Sun Yat-sen University, Guangzhou, China; 5 State Key Laboratory of Oncology in South China, Sun Yat-Sen University Cancer Center, Guangzhou, China

**Keywords:** Cancer therapeutic resistance, Oral cancer

**Correction to: Cell Death and Disease**

10.1038/s41419-019-2177-x published online 09 December 2019

Following publication of the article, the authors requested corrections to a grant ID in the acknowledgements section and to the label for Supplementary Fig. S3d.

The acknowledgement should read as follows:

“This work was supported by grants from National Natural Science Foundation of China (#81772892, #31801075), Science and Technology Program of Guangdong (#2016A030313348, #2018A030310344), Fundamental Research Funds for the Central Universities (#16ykjc17), The Key Laboratory of Malignant Tumor Gene Regulation and Target Therapy of Guangdong Higher Education Institutes, Sun-Yat-Sen University (Grant KLB09001), and Key Laboratory of Malignant Tumor Molecular Mechanism and Translational Medicine of Guangzhou Bureau of Science and Information Technology ([2013]163).”

In Supplementary Fig. S3d, the label “LDH release (%)” should read “Apoptosis Rate (%)”.

This has been corrected in both the PDF and HTML versions of the Article.

