# Supplementary material for: Vitamin D promotes the cisplatin sensitivity of oral squamous cell carcinoma by inhibiting LCN2-modulated NF-κB pathway activation through RPS3
Source: Cell Death Dis. 2019 Dec 9;10(12):936. doi: 10.1038/s41419-019-2177-x (PMC6901542; doi:10.1038/s41419-019-2177-x)
Supplement: Supplementary file 1 — Supplementary merged [file 41419_2019_2177_MOESM1_ESM.docx]

1. **Supplemental figures**


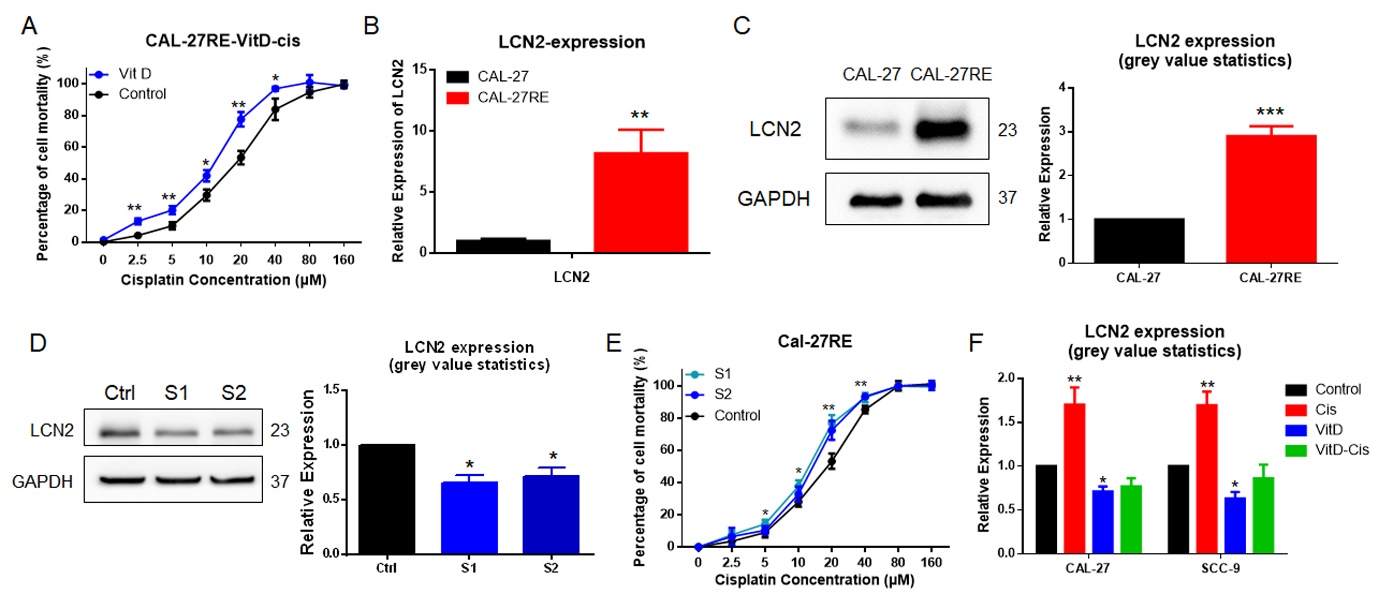


Figure 1S

A. MTS assays were performed to determine the sensitivity of CAL-27RE cells to gradient cisplatin after pretreatment with vitamin D;

B. LCN2 mRNA expression in CAL-27 and CAL-27RE cells;

C. LCN2 protein expression in CAL-27 and CAL-27RE cells;

D. Immunoblot verified that the siRNA successfully inhibited LCN2 protein expression in CAL-27RE cells;

E. Gradient concentration of cisplatin-treated siLCN2 CAL-27RE cells (MTS assays were performed to determine the inhibition of cisplatin sensitivity);

F. Grey value statistics analysis of Figure 2E.


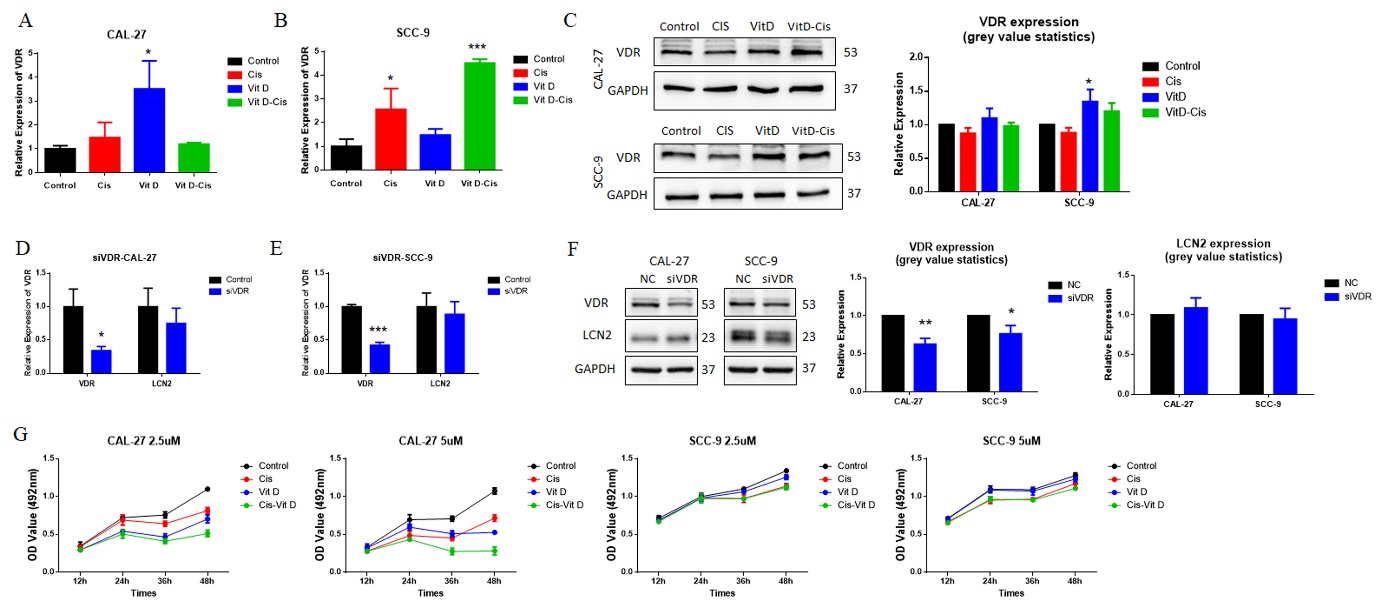


Figure 2S

A. VDR gene mRNA expression levels in CAL-27 cells after vitamin D and cisplatin treatment;

B. VDR gene mRNA expression levels in SCC-9 cells after vitamin D and cisplatin treatment;

C. VDR protein expression in CAL-27 and SCC-9 cells after vitamin D and cisplatin treatment;

D. VDR and LCN2 mRNA expression in CAL-27 cells 48 h after the downregulation of VDR;

E. VDR and LCN2 mRNA expression in SCC-9 cells 48 h after the downregulation of VDR;

F. VDR and LCN2 protein expression in CAL-27 & SCC-9 cells 48 h after the downregulation of VDR;

G. Effect of 30 nM vitamin D and 2.5,5 µM cisplatin on the proliferation of CAL-27 & SCC-9 cells.


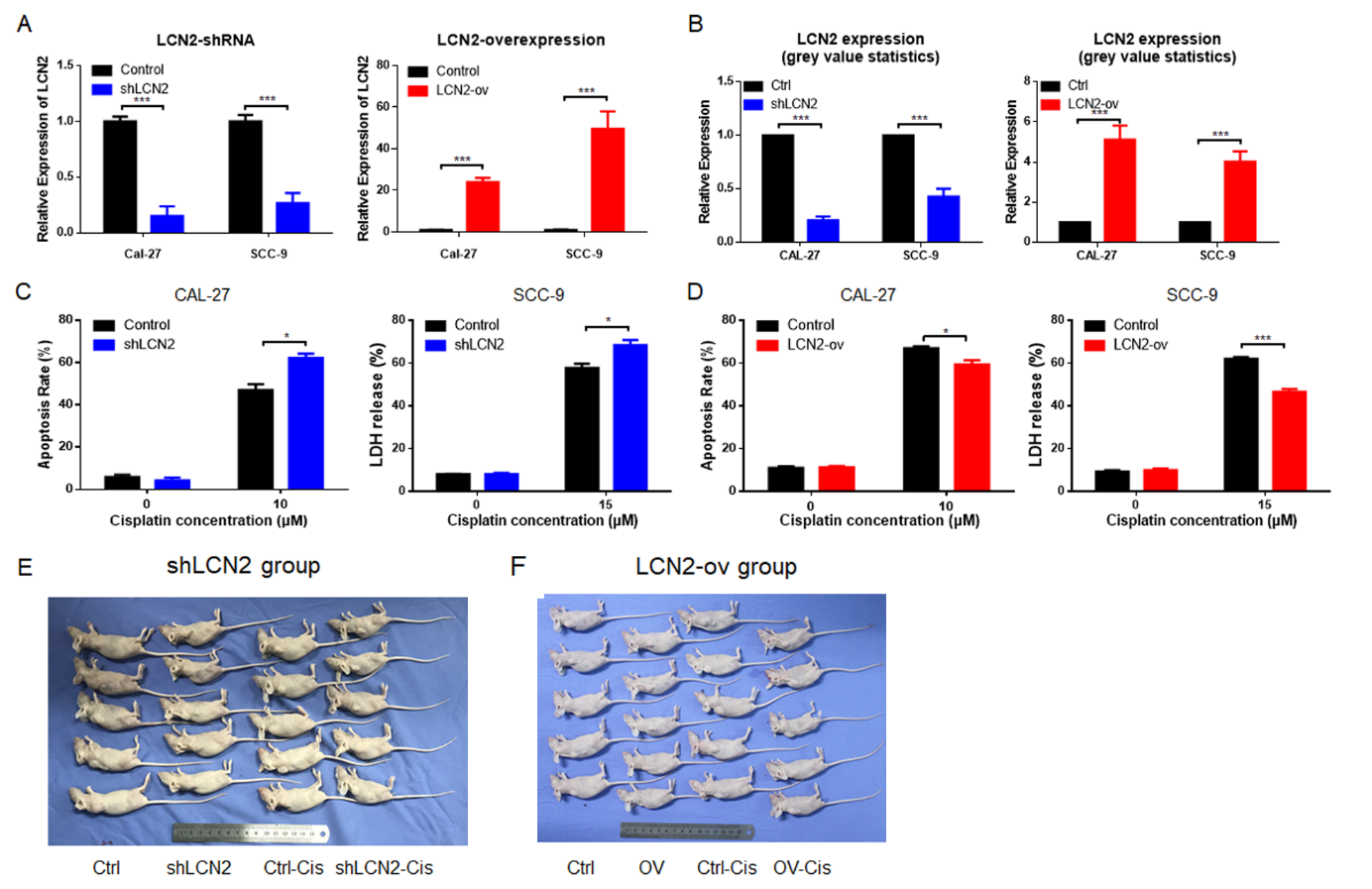


Figure 3S

A. qPCR confirmed that LCN2 was successfully inhibited or overexpressed in OSCC cells;

B. Grey value statistics analysis of Figure 3A.

C. Statistical results of flow cytometry shown in Figure 3D;

D. Statistical results of flow cytometry shown in Figure 3E;

E&F. Image of mice used in the xenograft experiments


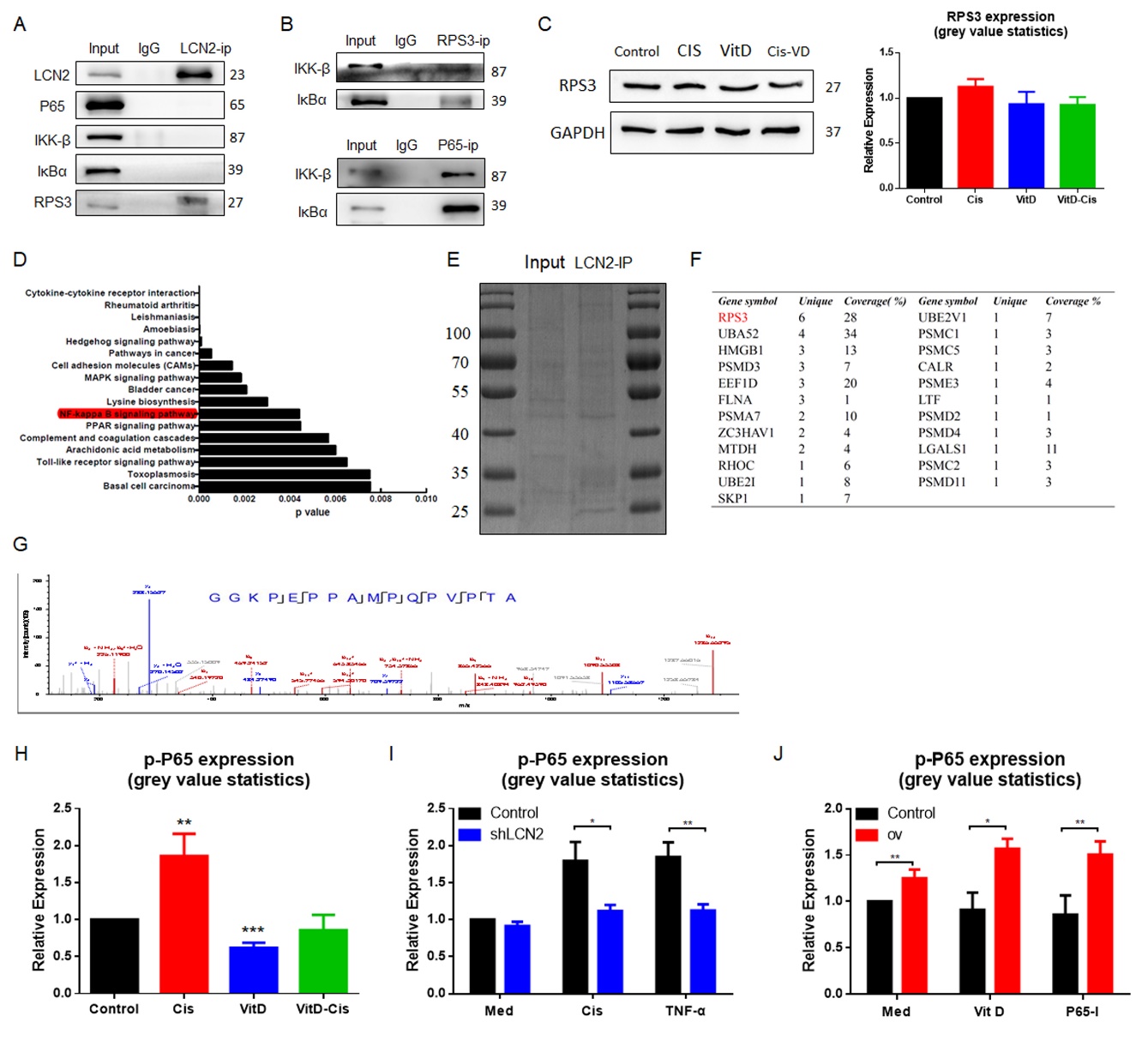


Figure 4S

A. Endogenous LCN2-IP assay: LCN2 did not show a significant interaction with the classical NF-κB signalling pathway but bound to RPS3;

B. Binding between IKK-β and IκBα in IP experiments on RPS3 and NF-κB;

C. Immunoblot analysis was used to detect the expression of RPS3 after vitamin D-cisplatin treatment;

D. KEGG analysis of RNA-seq results of CAL-27 cells showed cisplatin-induced genes were enriched in the NF-κB pathway;

E. IP assay using S-protein beads: silver staining revealed differential protein expression between the LCN2 and control groups;

F. Mass spectrometry analysis results of protein interaction with LCN2;

G. Mass spectral peptide results for RPS3;

H-J. Grey value statistics analysis of p-P65 in Figure 4A-C.


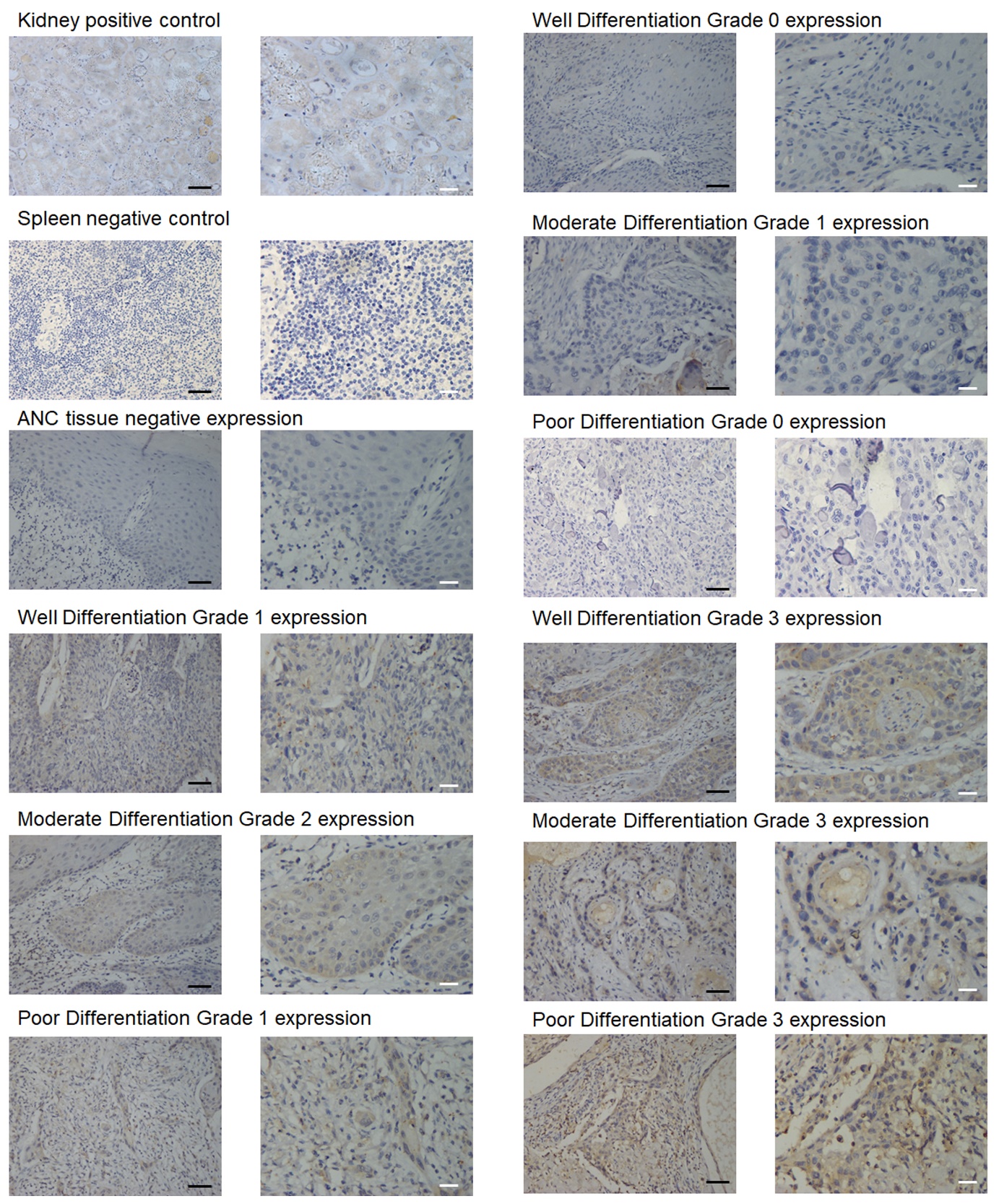


Figure 5S

Immunohistochemical analysis of LCN2 expression in different tissues (left 200x, scale bar, 50 μm; right 400x, scale bar, 20 μM)
