# Supplementary figures and images for: Vitamin D promotes the cisplatin sensitivity of oral squamous cell carcinoma by inhibiting LCN2-modulated NF-κB pathway activation through RPS3
Source: Cell Death Dis. 2019 Dec 9;10(12):936. doi: 10.1038/s41419-019-2177-x (PMC6901542; doi:10.1038/s41419-019-2177-x)

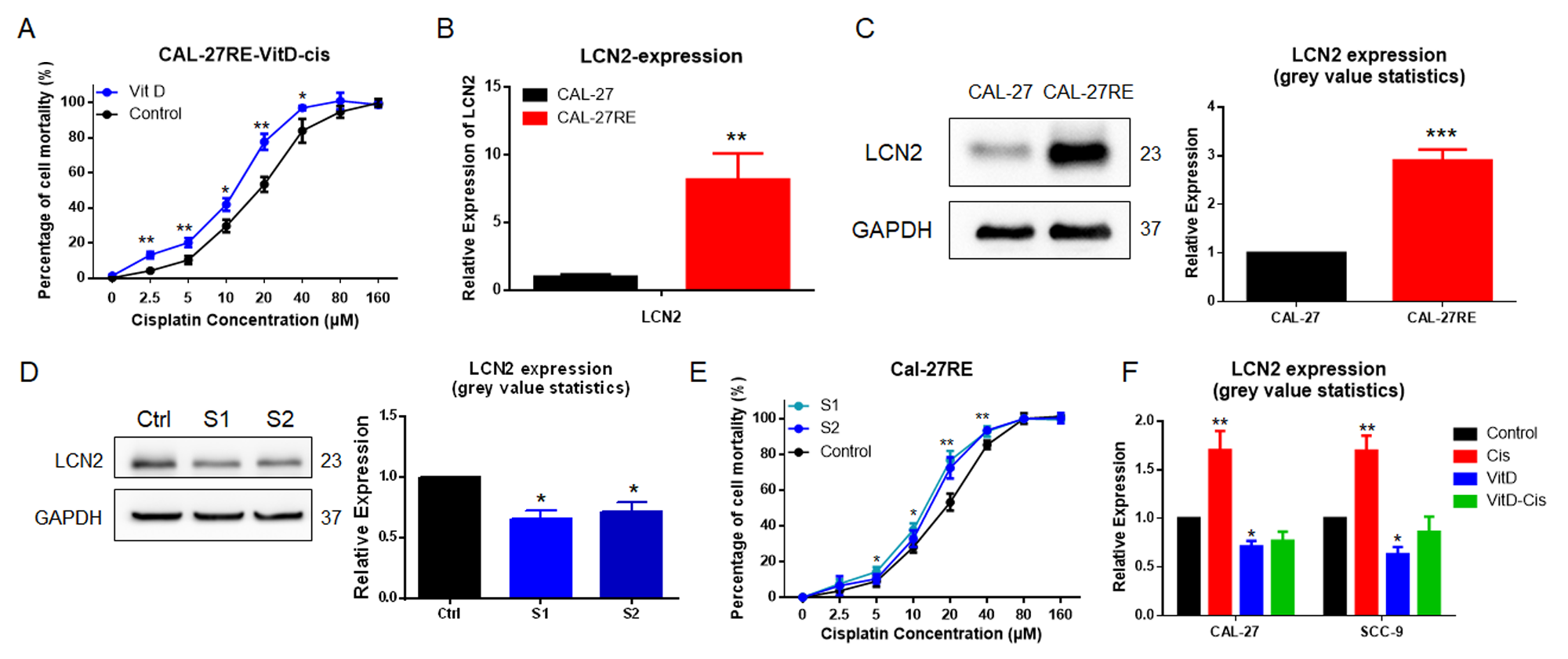

Supplement: Supplementary file 2 — Supplementary figure 1s [file 41419_2019_2177_MOESM2_ESM.tif]

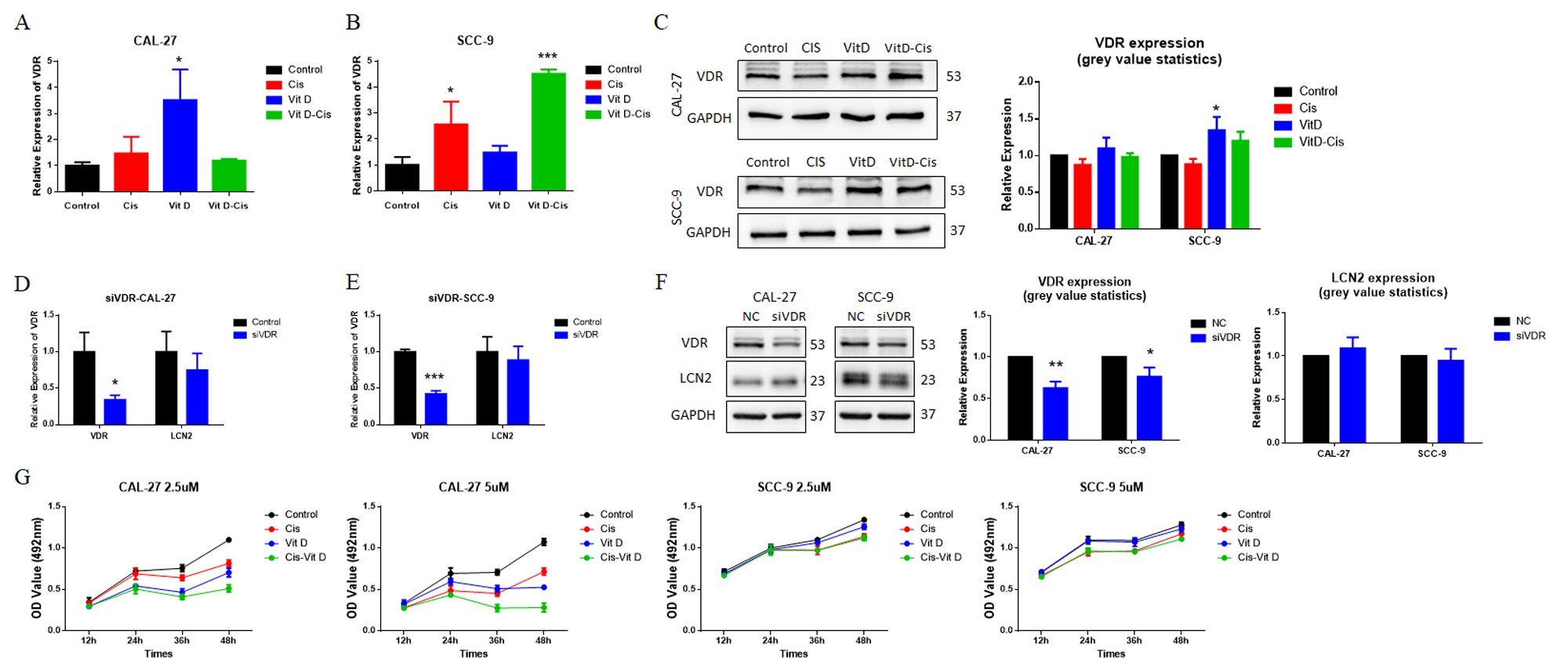

Supplement: Supplementary file 3 — Supplementary figure 2s [file 41419_2019_2177_MOESM3_ESM.tif]

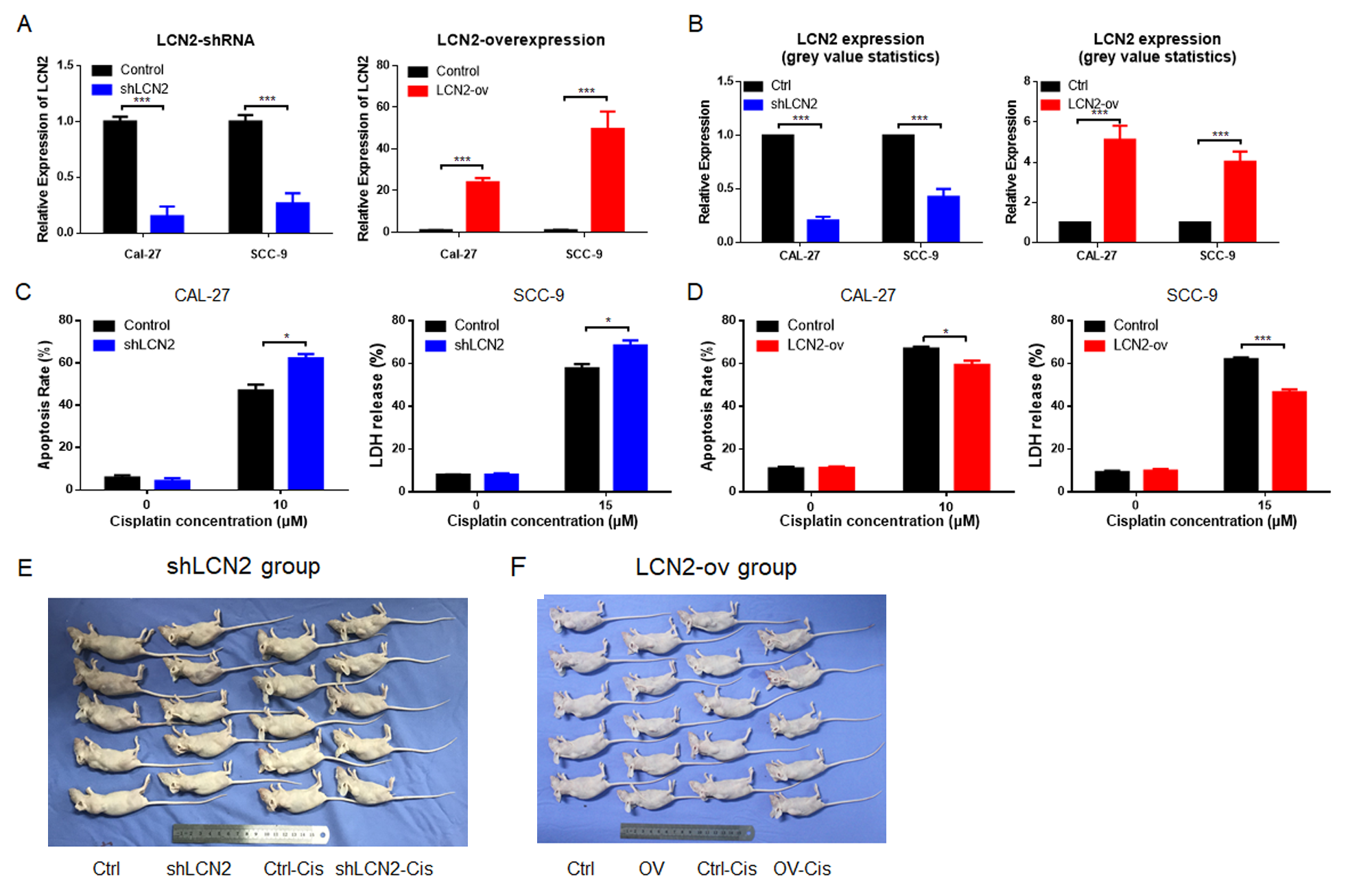

Supplement: Supplementary file 4 — Supplementary figure 3s [file 41419_2019_2177_MOESM4_ESM.tif]

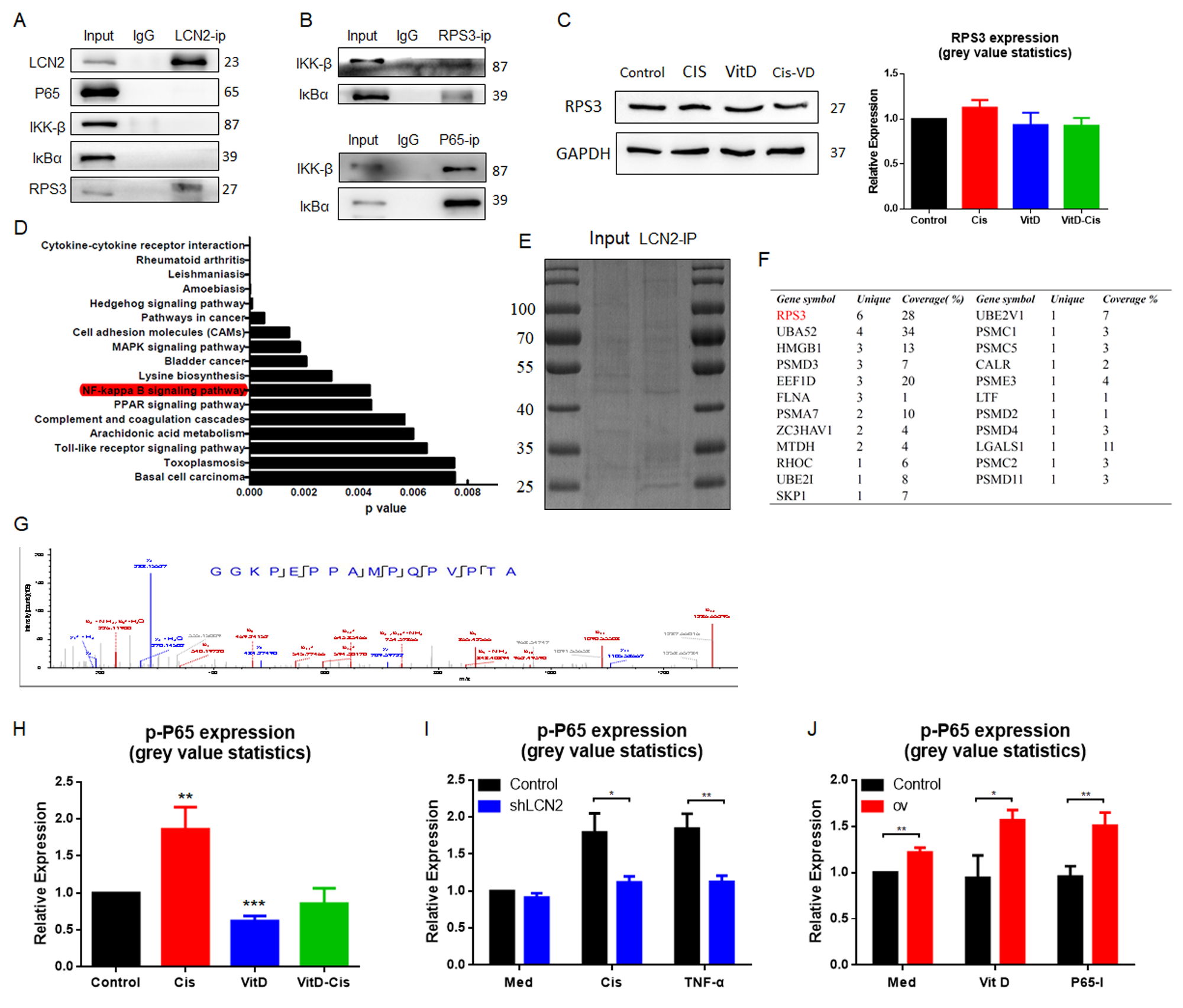

Supplement: Supplementary file 5 — Supplementary figure 4s [file 41419_2019_2177_MOESM5_ESM.tif]

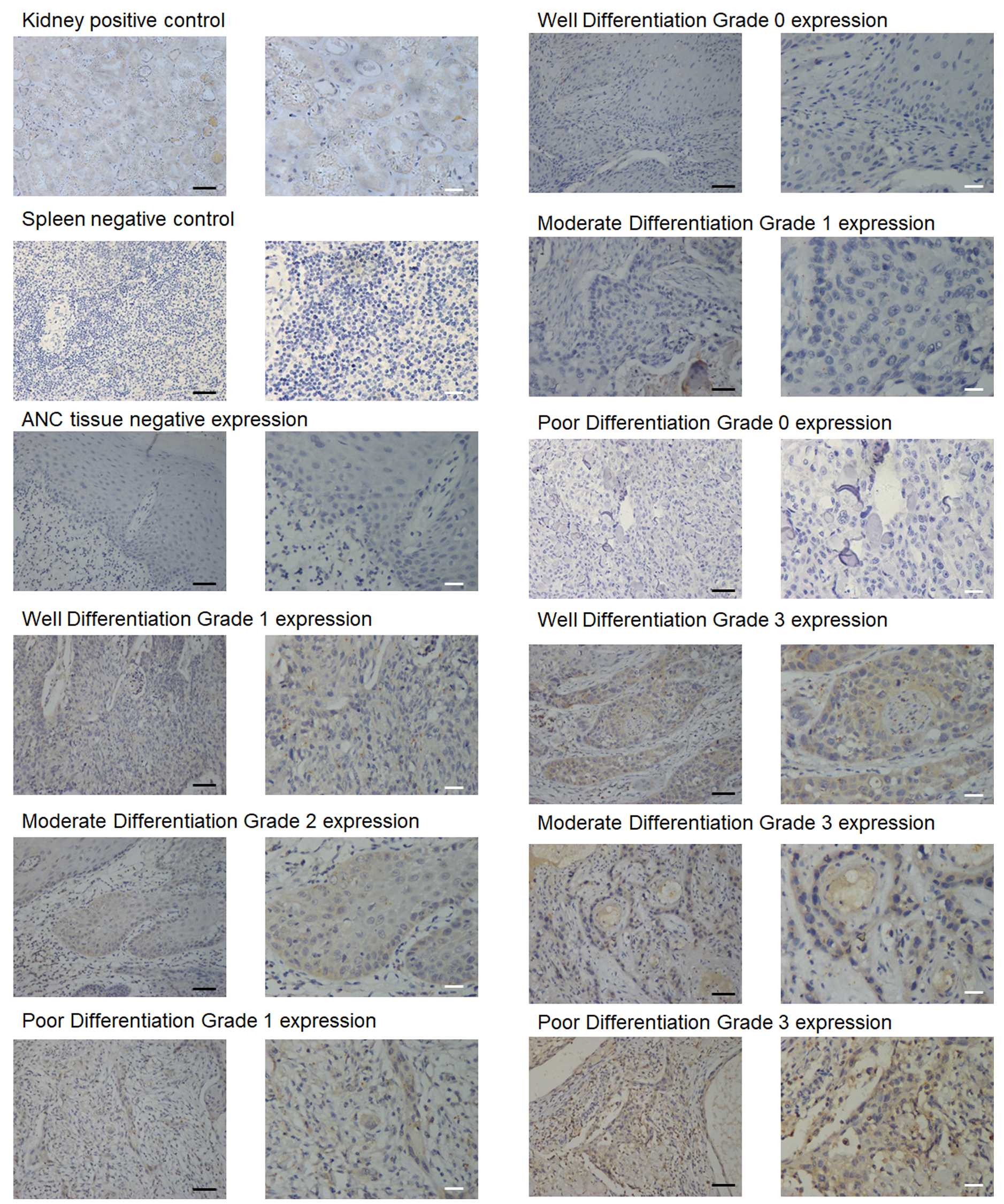

Supplement: Supplementary file 6 — Supplementary figure 5s [file 41419_2019_2177_MOESM6_ESM.tif]
